# Soft-assembled Multilevel Dynamics of Tactical Behaviors in Soccer

**DOI:** 10.3389/fpsyg.2016.01513

**Published:** 2016-10-05

**Authors:** Angel Ric, Carlota Torrents, Bruno Gonçalves, Jaime Sampaio, Robert Hristovski

**Affiliations:** ^1^Complex Systems in Sport Research Group, National Institute of Physical Education of Catalonia, University of LleidaLleida, Spain; ^2^CreativeLab Research Community, Research Centre in Sports Sciences, Health Sciences and Human Development, CIDESD, Universidade de Trás-os-Montes e Alto DouroVila Real, Portugal; ^3^Faculty of Physical Education, Sport and Health, Saints Cyril and Methodius UniversitySkopje, Macedonia

**Keywords:** exploratory behavior, interpersonal coordination, complex systems, collective variables, principal component analysis

## Abstract

This study aimed to identify the tactical patterns and the timescales of variables during a soccer match, allowing understanding the multilevel organization of tactical behaviors, and to determine the similarity of patterns performed by different groups of teammates during the first and second halves. Positional data from 20 professional male soccer players from the same team were collected using high frequency global positioning systems (5 Hz). Twenty-nine categories of tactical behaviors were determined from eight positioning-derived variables creating multivariate binary (Boolean) time-series matrices. Hierarchical principal component analysis (PCA) was used to identify the multilevel structure of tactical behaviors. The sequential reduction of each set level of principal components revealed a sole principal component as the slowest collective variable, forming the global basin of attraction of tactical patterns during each half of the match. In addition, the mean dwell time of each positioning-derived variable helped to understand the multilevel organization of collective tactical behavior during a soccer match. This approach warrants further investigations to analyze the influence of task constraints on the emergence of tactical behavior. Furthermore, PCA can help coaches to design representative training tasks according to those tactical patterns captured during match competitions and to compare them depending on situational variables.

## Introduction

In team sports settings, the interaction between team players and environment gives rise to interpersonal coordination movements that dynamically arise during the game. Interpersonal interactions are thought to be non-linear and some studies have shown this explicitly (Schmidt et al., 1990; Richardson et al., 2007). Under conditions of non-linear interpersonal interactions, behavioral patterns are hypothesized to be spontaneously organized at the macroscopic level as a result of functional grouping of components which are temporarily assembled, through a self-organization process (Gréhaigne et al., 1997; McGarry et al., 2002; Araujo et al., 2006). Many investigations have studied the emergence of coordination patterns on individual (Travassos et al., 2012), dyadic (Sampaio and Maçãs, 2012) and collective levels of analysis (Silva et al., 2014). Notwithstanding, there is little literature that attempts to identify the theoretically existing relation among these levels of game constraints (Bourbousson et al., 2014).

During soccer matches, there is no particular and fixed predetermined movement or social coordination to be executed for the achievement of a performance goal (score a goal) or sub-goal (avoid conceding one). Hence, there is a lack of convergence of the exploratory behavior toward a pre-defined intra- or inter-personal configuration. This situation allows very subtle and consequently unpredictable interactions between the environmental information flow and the performer's organismic constraints to decide which particular affordance, from the set of affordances, will be realized at each moment. Gibson (1979) originally used this term referring to opportunities for action. The interrelatedness of affordances (Bruineberg and Rietveld, 2014) allows us to understand the emergence of behavior at different levels of game constraints. Affordances offered by situational variables, such as quality or expertise of the opponent, can constrain ball possession strategies (Lago, 2009). In the same way, having ball possession far from or close to the opponent's goal typically constrains the emergence of specific tactical task solutions, for example, deep mobility of players while keeping the ball far from the opponent's goal, or, choosing a pressing strategy when the ball is lost in the opponent's field. All these interconnected situations are characterized by their evolution on different timescales (Mendes et al., 2007; Ric et al., 2016). This study of Ric et al. (2016) showed a clear separation of dynamical timescales in small-sided games. Slower evolution was characteristic of ball possesion or dispossesion and quicker evolution was characteristic for specific actions belonging to the former states. This state of affairs clearly reflects the model-independent definition of dynamic property of metastability^1^ as a property related to the existence of multiple separated timescales. At quick timescales, the system appears to be in equilibrium, i.e., in a temporarily stable state, but explores a limited part of its available state space. At longer timescales, however, it undergoes transitions between such metastable states (Bovier and Den Hollander, 2016). These previous studies allow us to hypothesize that the characteristic timescales of team behaviors could define the interpersonal coordination at different levels of system organization and help to understand the nested structure of the dynamics of tactical behaviors.

The dynamical systems approach has established the bases for understanding the emergence of interpersonal coordination in team sports requiring the identification of relevant coordination variables [described as collective variables or modes and their amplitudes, i.e., order parameters (Haken, 2006)]. These collective variables describe the spatiotemporal pattern of interpersonal coordination and the changes of coordination (Riley et al., 2011) that occur in response to game constraints. In a team sport setting, the relative phase between two oscillating components has been frequently used as a collective variable to capture the macroscopic order or coordination of the dyadic system. Bourbousson et al. (2010), through the relative phase between the oscillations of mean player dispersion around the team geometrical center (i.e., stretch index) of two confronted basketball teams, found that team players tended to expand and contract together when both teams were moving from basket to basket showing clear inter-team coordination patterns. Travassos et al. (2011) showed that interpersonal coordination between defenders was stronger than between attackers demonstrating that attacking players show greater variability in the movement patterns performed during futsal matches. Gonçalves et al. (2014) applied relative phase analysis between the mean position of specific group players (i.e., team geometrical center) over time during a soccer match. They found that stronger coordination movements appeared between the consecutive line forces, that is, defender-midfielders and midfielders-attackers. Other techniques, commonly used to capture coordination patterns, like cross-correlations and vector coding, have recently been studied (Moura et al., 2016).

Whereas these studies focused on the coordination between two oscillating variables, principal component analysis (PCA) has been successfully proposed as a multivariate statistical method for analyzing high dimensional (i.e., >2) movement coordination patterns (Daffertshofer et al., 2004; Forner-Cordero et al., 2005). In essence, PCA reduces the dimensionality of large data sets, obtaining a smaller number of subjacent components that explain most of the variance and summarizes the information of the original variables. PCA has been used in the past three decades as an efficient method of defining the essential, i.e., collective, variables in a range of complex dynamical processes such as protein folding (Matsunaga et al., 2007; Hayward and De Groot, 2008; Maisuradze et al., 2009) and brain dynamics (Jirsa et al., 2002). In sport science, PCA has allowed identifying the performance of an interpersonal precision task (Ramenzoni et al., 2012) to determine the impact of different training programs on cardio-respiratory coordination (Balagué et al., 2016), and to identify different dancing coordination patterns depending on concrete task constraints (Bronner and Shippen, 2015; Torrents et al., 2015). In soccer, PCA has been applied to capture player positional patterns and their variability (Barros et al., 2006). Moura et al. (2015) used this method by applying it to each mean player position in the field in order to obtain the collective organization during the matches of subsequent rounds in the European Championship and the positional variability of all players. Despite these investigations, the use of PCA to detect the pattern-forming dynamics on a collective level over the course of a competitive match and the relation between the adjacent levels of game constraints remains unexplored. In this sense, several authors have emphasized the need to analyze the timescales of tactical behavior that unequivocally define the multi-level game dynamics because nested levels in dynamical systems are inevitably connected with characteristic timescales of their evolution (Haken, 2006). Therefore, the aim of this study was to identify the tactical patterns and the timescales of positioning-derived variables that define the patterns during a soccer match, allowing understanding the multilevel organization of tactical behaviors.

## Materials and methods

### Participants and procedure

Twenty male professional soccer players from the same team (age = 22.8 ± 4.4 years; professional playing experience = 6.9 ± 4.1 years) participated in an official pre-season match across the two halves against another team. Goalkeepers participated in the match but were excluded from the analysis. Due to the characteristics of pre-season matches, the ten starter outfield soccer players participated in the first half. Eight of them were substituted at halftime and the other two in the middle of the second half in order to ensure a more constant team formation and avoid the effects of cumulative fatigue (Mohr et al., 2005). The match was played on a natural turf pitch (100 × 68 m) following the official soccer rules. The players analyzed belonged to the visiting team. The final score of the match was 1-0. All players provided written informed consent to participate in the experiment. The local institutional Research Ethics Committee approved the study, which also conformed to the recommendations of the Declaration of Helsinki.

### Data collection and preparation

Positional data from the outfield players were collected using 5 Hz GPS devices (SPI Pro, GPSports, Canberra, Australia). Each one was placed on the upper back of the players. Latitude and longitude coordinates were exported from the units and computed using dedicated routines in Matlab R2014b software (MathWorks, Inc., Massachusetts, USA; for complete guidelines, see Folgado et al., 2014).

Configurations of tactical behaviors expressed in team-related positioning-derived variables were computed to determine the structural and dynamic characteristics of the team. Eight collective measures were processed from the outfield players: stretch index, team length, team width, longitudinal position of team geometrical center (x axis) and lateral position of team geometrical center (y axis) (Duarte et al., 2013). Whereas team geometrical center (also named centroid) and stretch index have been previously defined, team length, and width were calculated as the difference between the maximum and minimum positions of players in the field's longitudinal and latitudinal dimensions, respectively, in each time unit. Also included was the speed of displacement (meters per second) at which team geometrical center was moving (differentiated lateral and longitudinal axes) and the speed of contraction and expansion in order to capture how the team was behaving (Bourbousson et al., 2010). This last variable was calculated by differencing the each data point of the stretch index with the previous one.

Data were down sampled into 1 Hz in order to define the team configuration at each second. A two-step cluster analysis was performed to determine automatically the boundary values of each positioning-derived variable. To determine the zone around which the geometrical center was located, the field was divided into four sectors and three corridors (Costa et al., 2011; Sarmento et al., 2013). Finally, 29 categories were determined to create multivariate binary (Boolean) time series matrices (Table 1). A value of 1 was ascribed to the active categories and value of 0 to the inactive ones, representing the full configuration (tactical pattern) during the same time interval of 1 s. Each 1-s window was defined as a 29-component binary vector (column). A total of 2700 configurations (45 min × 60 s) for each half were finally obtained.

**Table 1:** **Categories of each collective positioning-derived variable with the boundary values, identifying a total of 29 categories**.

1–2: Stretch index	< 16.75 m	Small	SIS
	>16.75 m	Large	SIL
3–6: Speed of spread	< −0.6 m/s	Quick contraction	QC
	−0.6 to 0 m/s	Slow contraction	SC
	0–0.6 m/s	Slow expansion	SE
	>0.6 m/s	Quick expansion	QE
7–10: Length	< 27.72 m	Small	LS
	27.72–35.4 m	Medium	LM
	35.4–42.71 m	Large	LL
	>42.71 m	Very large	LVL
11–14: Width	< 34.49 m	Small	WS
	34.49–43.68 m	Medium	WM
	43.68–53.59 m	Large	WL
	>53.59 m	Very large	WVL
15–18: Sector	< 25 m	Ultra-defensive	UDS
	25–50 m	Mid-defensive	MDS
	50–75 m	Mid-offensive	MOS
	>75 m	Ultra-offensive	UDS
19–21: Corridor	< 21.33 m	Right	RC
	21.33–42.66	Central	CC
	>42.66	Left	LC
22–25: Longitudinal speed of team center	< −1.06 m/s	Quick drop back	QDB
	−1.06 to 0 m/s	Slow drop back	SDB
	0–1.06 m/s	Slow forward move	SFM
	>1.06 m/s	Quick forward move	QFM
26–29: Lateral speed of team center	< −0.6 m/s	Quick to the right	QR
	−0.6 to 0 m/s	Slow to the right	SR
	0–0.6 m/s	Slow to the left	SL
	>0.6 m/s	Quick to the left	QL

### Data analysis

#### Principal component analysis

Hierarchical PCA (hPCA) was performed to define the collective (state) variables of the coordinated behaviors of teams on separated levels of organization. The higher levels were hypothesized to be dominantly defined by categories that possess larger dwell times. The initial data were multivariate binary matrices: 29 categories × 2700 time-ordered configurations (for the suitability of using principal components analysis with binary variables see Joliffe, 2002). The initial system of PCs was rotated under the Direct Oblimin method with δ = 0 (Westerhuis et al., 1998) to consider the possibility of a higher-order structure in data because of the correlation between the extracted components in each order. The number of significant principal components (PCs) was determined by the Kaiser-Gutmann criterion but only those that cumulatively accounted for ≥80% of the explained variance were further selected (Fabrigar et al., 1999).

The hPCA differs from the ordinary PCA in that it is free from the assumption of orthogonality of the principal components. In other words, it is based on more general assumptions of non-orthogonality of correlation/covariance matrix eigenvectors (PCs) and treats orthogonality as a special case. Just as the ordinary PCA is based on reducing the original high dimensional data to a lower dimensional space based on correlations/covariance of observed data, the hPCA treats the correlations of the extracted first order PCs as a new input to further reduce the dimensionality to fewer and higher-order PCs. The procedure continues until the moment when no further meaningful correlations/covariance between the PCs is detected. If the first order PCs share little meaningful variance, the results of hPCA are comparable to the orthogonal PCA solutions. Principal component scores (see Joliffe, 2002) were used to determine the most salient categories which defined each principal component (i.e., tactical pattern). Principal component scores were estimated as: *C* = *R*^−1^ *S*, where *R* is the correlation matrix of time-ordered game configurations and *S* is the PC structure matrix, i.e., the correlations between 2700 time-ordered game configurations and PCs (Fulgosi, 1988).

The structure matrix was used to visualize the dynamics of team configurations in the space spanned by the extracted PCs. Finally, to compare the structure of first-level PCs between both halves, Tucker's congruence coefficient was used to determine the degree of similarity between principal components (Lorenzo-Seva and ten Berge, 2006).

#### Analysis of timescales of positioning-derived variables

The aim of the analysis was to identify the dynamic properties of the game assessed by the associated dwell (waiting or residence) times of positioning-derived variables. Dwell times assess how long a certain variable remains in a well-defined state before leaving it and switching to another. Hence, they are useful in this respect since their averages show the speed of evolution of the variable in question. The shorter the average dwell time the quicker the evolution (changing the states) and vice versa. The pooled averages of the active categories (i.e., with 1 ascribed) were calculated in order to find out the average time, in seconds, that the team was dwelling on each positioning-derived variable.

In addition, the video-recorded match was analyzed to calculate the timescale (i.e., average dwell time) on which ball possession switched from one team to another. The beginning of ball possession started when: the goalkeeper took the ball in his hands, the second touch of the player winning back the ball, or the first touch of any teammate after a pass, deflection, or clearance by the teammate who first touched the ball (Castellano, 2008). It is important to note that when play was stopped due to an interruption (e.g. corner kick, fouls, goals, etc.) ball possession was assigned to the team responsible for restarting the game.

Due to non-Gaussian distributions of the dwell times, the non-parametric Kruskal-Wallis test was performed in order to compare dwell times of variables to identify possible slow- and fast-evolving processes. It was conducted to compare the timescales of all positioning-derived variables between both halves. The partial eta square (*p*η^2^) value is reported as a measure of effect size and is interpreted according to the following criteria: significant but weak (ES ≤ 0.04), moderate (0.04 < ES ≤ 0.36) and strong (ES > 0.36) (Tabachnick and Fidell, 2007). Comparisons between halves were assessed via standardized mean differences, computed with pooled variance and respective 90% confidence intervals. Dwell time data were log-transformed to reduce bias arising from a non-uniformity error. Uncertainty in the differences was expressed as 90% of confidence limits (CL) and as probabilities that the true effect was substantially greater or smaller than the smaller practical difference at the threshold of 25% (declared possible). These probabilities were used to make a qualitative probabilistic mechanistic inference about the true effect. The scale was as follows: 25−75%, possible; 75−95%, likely; 95−99%, very likely; >99%, most likely. A difference was assessed as being unclear if the CI overlapped both substantially positive and negative thresholds by ≥5%. The Cohen d effect size with 90% CL was calculated using pooled standard deviation for comparisons and the magnitude ranges for mean differences were: 0–0.2 trivial; > 0.2–0.6 small; > 0.6–1.2 moderate; > 1.2–2 large; > 2 very large (Hopkins et al., 2009).

## Results

### The primary level of tactical patterns

**Figure 1:**
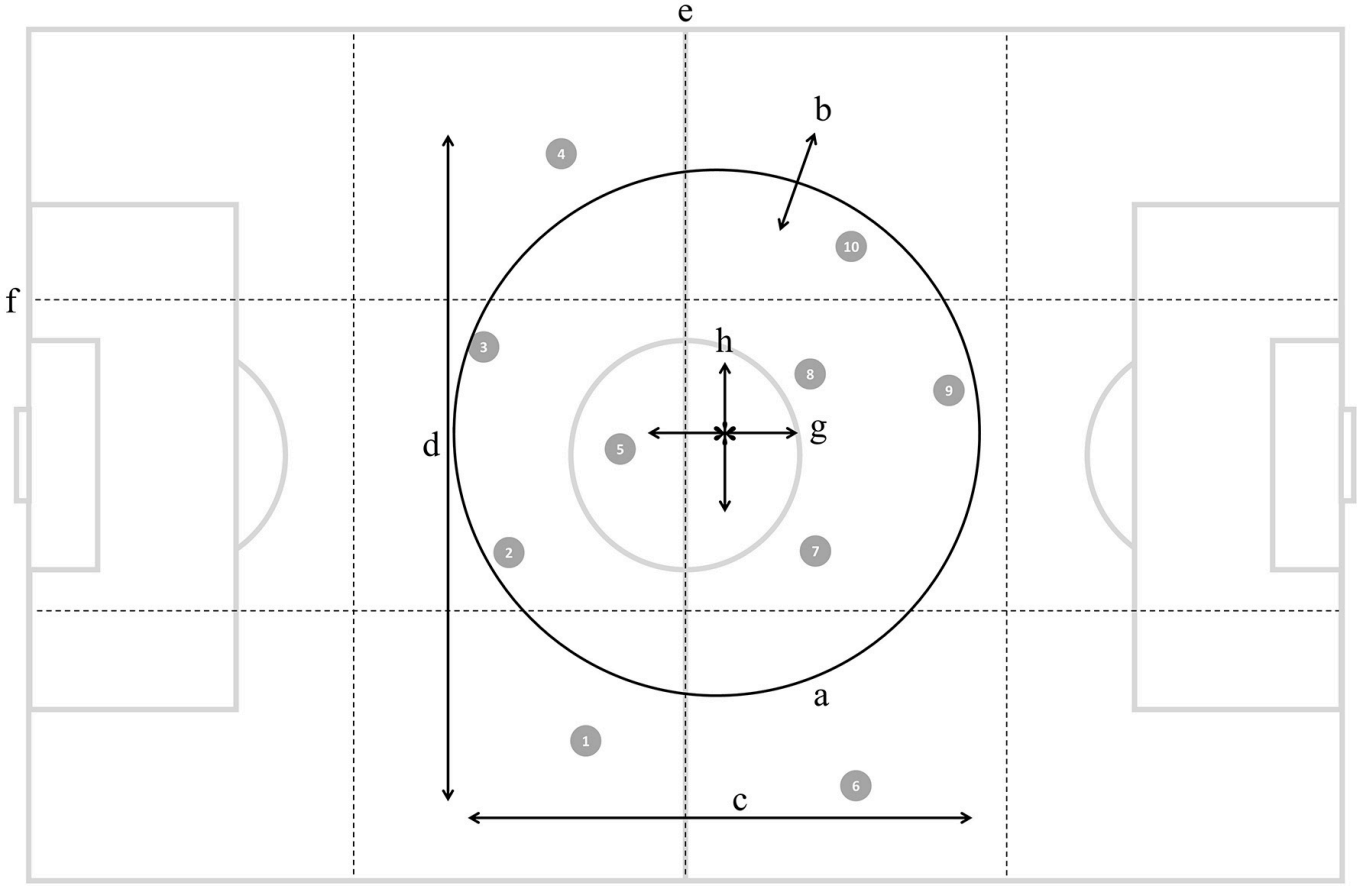
**Illustration of ten outfield players (numbered gray circles) and the eight positioning-derived variables: (a) stretch index, (b) speed of dispersion, (c) length, (d) width, (e) sectors, (f) corridors, (g) longitudinal speed of team center, and (h) latitudinal speed of team center**.

PCA initially revealed 22 principal components. Twelve of these represented 80.8 and 80.91% of the total variance for the first and second half, respectively. The high positive and negative component scores on these twelve principal components represented those tactical variables (categories) that occurred and decayed jointly (Figure 1). The salient structure of PCs was defined by those categories with a high absolute (positive or negative) component score. Scores close to 0 indicate that the corresponding category does not contribute or hardly contributes to the PC. High positive scores (>1) informs that those categories are simultaneously active while high negative scores (< − 1) report that the corresponding categories are active together but are active when the positive ones are inactive and inversely.

**Figure 2:**
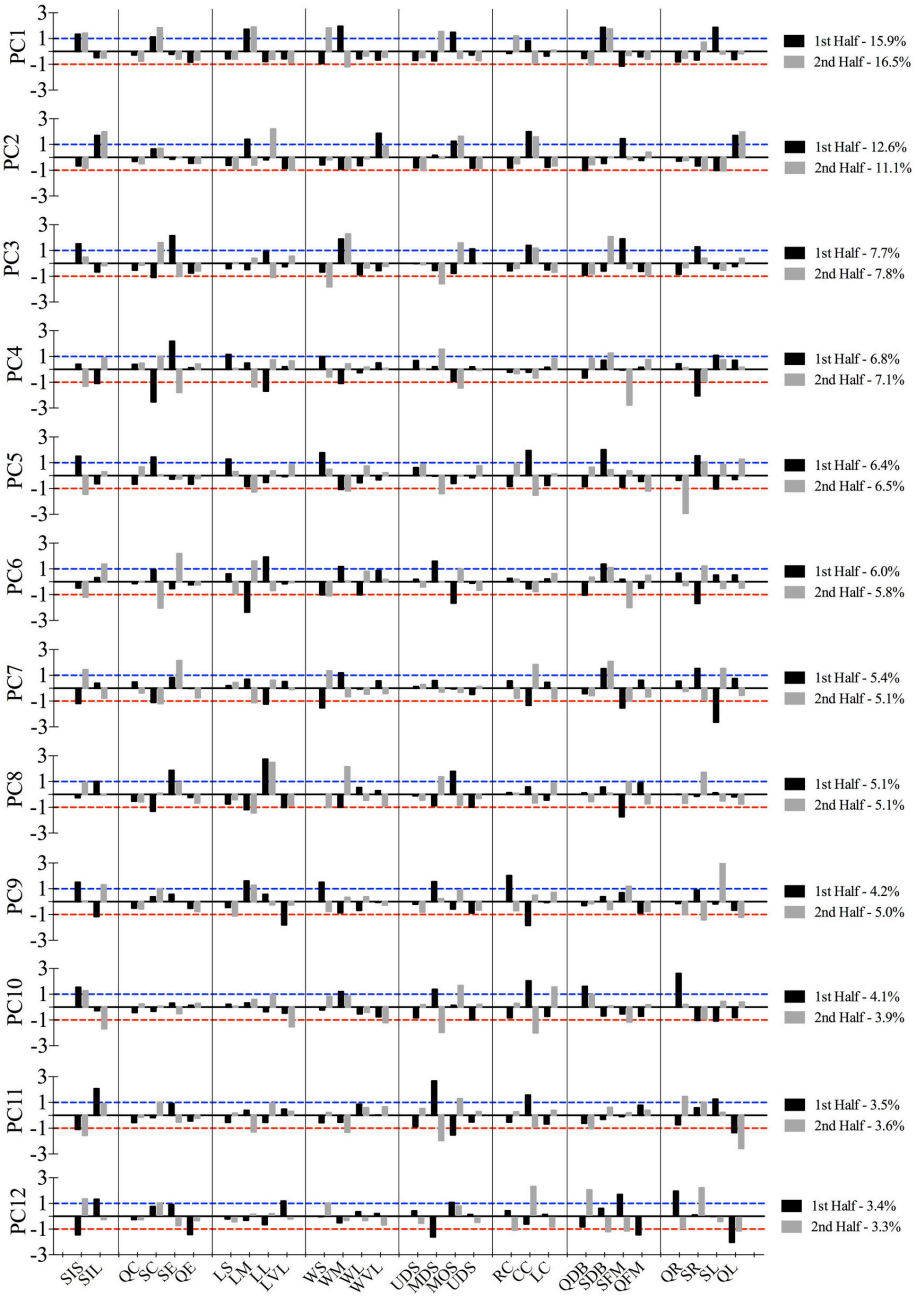
**Larger values than 1 (blue dashed line) and/or lower than −1 (red dashed line) refer to the tactical behaviors that defined the primary-level of principal components**.

The structures of PCs showed significant congruence between PC1 of the first half and PC3 of the second (*r*_*c*_ = 0.70). They can be defined as defensive patterns because of the medium width and small stretch index which slowly shrank, with the team centroid located in middle offensive sector and central corridor while they were dropping back (see Figure 2). The percentage of the total variance that explained this pattern in the first part was twice that of the second. These patterns could describe the main positioning structure when they were defending in the first half. On the other hand, the most frequent tactical pattern in the second half (PC1) had a significant degree of similarity (*r*_*c*_ = 0.78) with PC9 of the first half. They are defined as defensive patterns and were characterized by a small stretch index, related to the medium length and small width, with the team centroid located in the middle defensive sector and right corridor. The players were slowly reducing their effective playing space and dropping back in PC1 but this was not clearly defined in PC9. Defending patterns were the most stable patterns in both halves, but, whereas in the first half the team was located in the opponent's field for defending, in the second they were placed mostly in their own field. The congruence coefficient between PC2 in both halves showed a significant similarity between them (*r*_*c*_ = 0.73). These offensive patterns were defined by a large stretch index, with the team keeping the distances between the players and their geometrical center mostly stable. The team was moving forward and quickly to the left, with the team centroid located in central offensive sector and central corridor. The third PC of the first half was defined by a small but slowly increasing dispersion of players, with a long length and medium width. Besides, the location of the team center was around the ultra-offensive sector and it was slowly moving forward to the right showing the dominance of the game in the opponent's field. Its highest congruence value (*r*_*c*_ = 0.61) is related with PC8 of the second half, explaining two similar offensive patterns for each half. In addition, PCs also showed significant congruence coefficients: PC12 of the first half with PC11 of the second (*r*_*c*_ = 0.70) PC11 with PC10 (*r*_*c*_ = 0.76) and PC10 with PC5 (*r*_*c*_ = 0.79). The rest of combinations did not reach coefficients of congruence higher than 0.70, however their structure can be consulted in Figure 1.

### Metastable dynamics of tactical patterns

**Figure 3:**
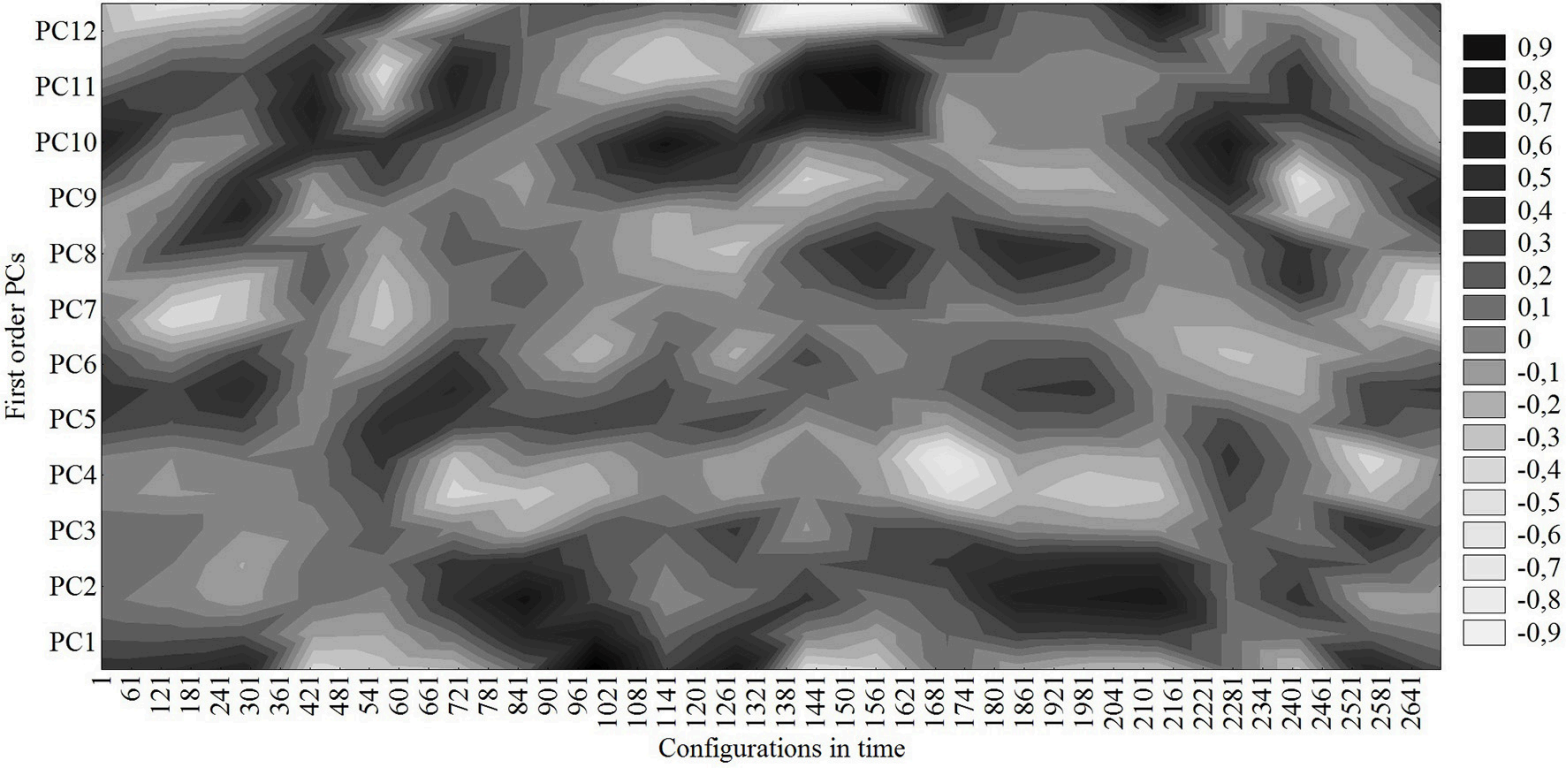
**Correlations (loadings) of time-ordered game configurations with the first-level PCs**.

Figure 3 represents the loadings (correlations) of the time configuration vector on/with the PCs. Note that the configuration vector dwells for some time projecting dominantly on a few PCs and then, on a longer timescale, quickly transits to form another temporarily stable set of dominant projections. These sets of dominant projections of configurations on the PCs represent the attractors of the team dynamics. The dominance of projections refers to the dominant PC content of the configurations, while the dwell time of the dominant projection to its stability or attraction strength (the longer the dwell time the greater the stability or attraction strength and vice versa).

As can be seen in Figure 3, during the first 5 min the system was projecting mostly on PC1, PC5, and PC9 defined by a small stretch index that slowly contracted and team positioning was slowly dropping back. Although the locations of the team center were in different sectors, all these patterns were defensive. Then, the switches between these PCs at the beginning of the match were brought about by the small differences in some of the categories that defined each one of these PCs. It is interesting to note that the dominant projection of the game configuration when the goal was scored (second 1148) corresponds to PC10. The small dispersion of players was not altered much more, but the team center quickly moved back to the right side from the central corridor of the middle defensive sector. That pattern clearly defines the defense of the opponent's counterattack. After the goal, the game configuration transited to a dominant projection on PC11 defined by the large dispersion, slowly growing up from the middle of the field quickly moving to the opponent's midfield. Finally, the team was mostly stable performing PC2, previously defined, for 7 min before transiting to another less stable tactical pattern.

### Multilevel organization of tactical patterns

**Figure 4:**
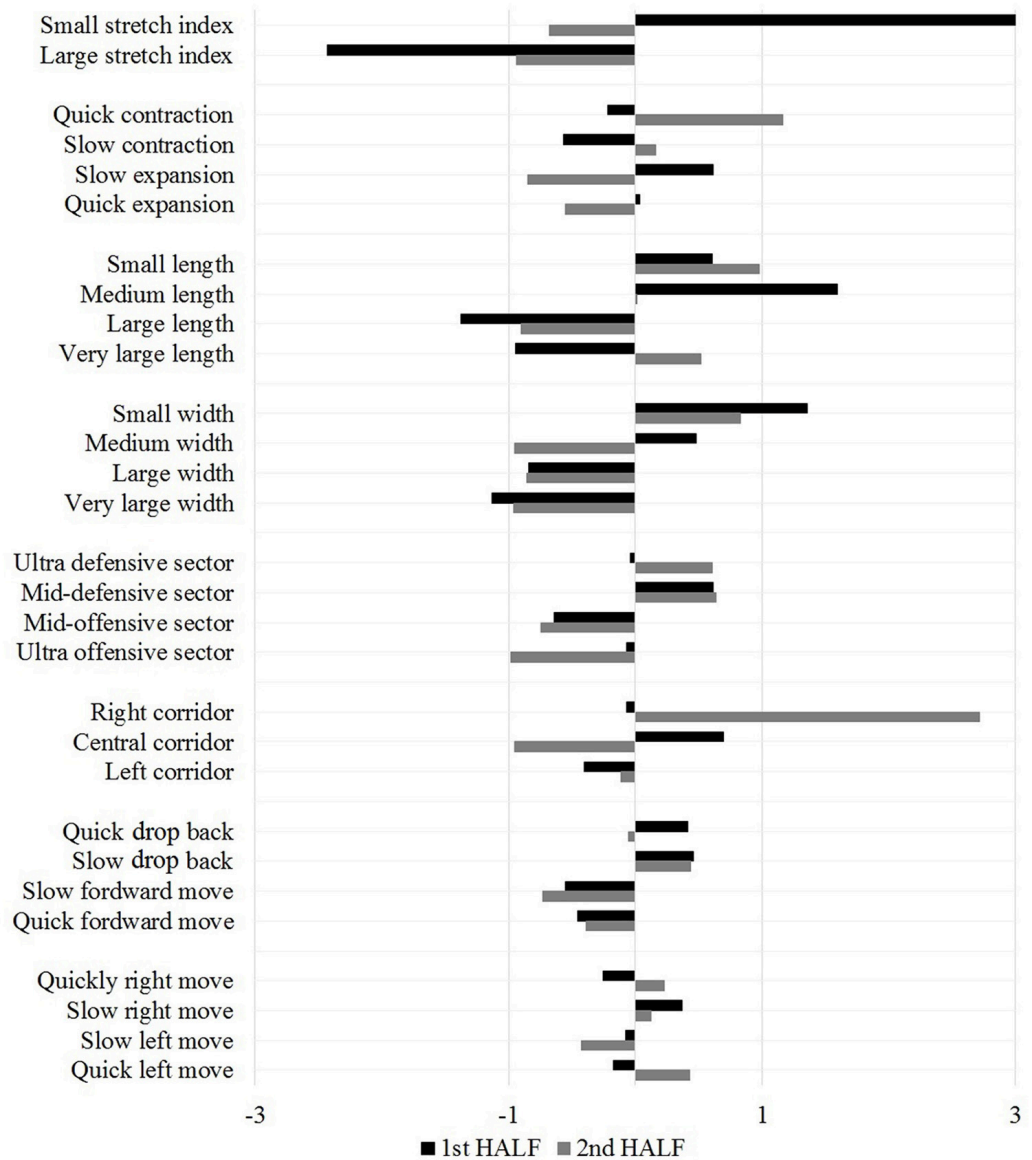
**Component scores of the highest level PC for the first and second half**.

First-level PCs correlations were then subjected to a further higher-order analysis revealing second-level PCs. Four PCs in the first half of the match and five in the second half were identified. Furthermore, the second-level correlated structures systematically produced a sole PC on the third-level for the first half (see black bars in Figure 4), whereas two PCs were extracted from the second half. Therefore, by a further iteration of the procedure a fourth-level PC was extracted only in the second half (see gray bars in Figure 4). The lower-order PCs are more sensitive to detailed changes in impinging game constraints, while the highest-level PC (third-level PC in the first half and fourth-level PC in the second) captures the most robust and stable structure of associations within the data, defining the most persistent patterns over time. The congruence coefficients of these highest-level PCs reached a value of 0.69.

The Kruskal-Wallis test showed a significant effect between the timescales of all positional variables including ball possession: [H (8, *N* = 3156) = 845.8; $X_{\left(8\right)}^{2}$ = 513.51; *P* < 0.0001; *p*η^2^ = 0.314] for the first half and [H (8, *N* = 3257) = 911.66; $X_{\left(8\right)}^{2}$ = 527.63; *P* < 0.0001; *p*η^2^ = 0.324] for the second. Table 2 shows means and standard deviations of timescales for each variable differentiating both halves. The probability of finding true differences between halves was possible for timescales of stretch index and ball possession, with a positive and negative effect, respectively. An unclear tendency was reported for the dwell time of the centroids allocated in the corridors. Finally, trivial-to-unlikely differences between the first and second half were found in the timescales for the remaining collective positional variables.

**Table 2:** **Mean values ± ***SD*** for different collective positional variables and ball possession and corresponding difference comparisons between first and second half**.

	**Mean** ± ***SD*** **(*****n*****)**		**Differences observed for the first half compared with second**			
	**First half**	**Second half**	**Difference in means (%)**	Chances^*^	**Qualitative assessment**	**Effect size**
Stretch index	23.94 ± 22.70 (111)	26.44 ± 25.96 (102)	15.36; ± 25.97	34/65/1	Possibly ↑	0.14; ± 0.23
Speed of dispersion	3.37 ± 3.32 (800)	3.24 ± 2.93 (833)	−2.48; ± 6.08	0/100/0	Most likely trivial	−0.03; ± 0.08
Length	7.27 ± 7.56 (370)	7.55 ± 8.46 (357)	2.12; ± 11.21	1/99/0	Very likely trivial	0.02; ± 0.12
Width	9.20 ± 9.10 (289)	9.27 ± 9.52 (291)	0.18; ± 12.57	1/98/1	Very likely trivial	0.00; ± 0.14
Sectors	22.05 ± 18.21 (119)	24.37 ± 25.31 (110)	−3.27; ± 22.53	4/86/10	Likely trivial	−0.03; ± 0.22
Corridors	37.10 ± 47.07 (73)	29.96 ± 32.69 (90)	−0.68; ± 33.40	10/79/11	Unclear	−0.01; ± 0.26
Longitudinal speed of team center	4.50 ± 5.03 (598)	3.98 ± 3.96 (678)	−5.34; ± 7.71	0/99/1	Very likely trivial	−0.06; ± 0.09
Lateral speed of team center	3.82 ± 3.84 (707)	3.89 ± 3.96 (693)	1.38; ± 7.67	0/100/0	Most likely trivial	0.02; ± 0.09
Ball possession	30.30 ± 32.30 (89)	26.19 ± 25.91 (103)	−11.02; ± 22.82	2/72/27	Possibly ↓	−0.11; ± 0.24

* *Percentage chance of having substantially positive/trivial/substantially negative effect*.

*↑, Increase; ↓, decrease*.

Multiple comparisons between the means of all eight variables (see Table 3) allowed identifying three groups of variables that possessed different characteristic timescales. Those variables that evolved over the timescales of tens of seconds, i.e., stretch index, sectors, and corridors, showed statistical differences with all other variables, whereas no significant differences were detected when compared between them (stretch index vs. sectors *p* = 1.00; stretch index vs. corridors *p* = 1.00; sectors vs. corridors *p* = 1.00 for the first half and *p* = 1.00; *p* = 1.00; *p* = 1.00 for the second). Similarly, significant differences were absent in comparisons of those variables that quickly evolved, i.e., over the timescale of a few seconds. For the first half: speed dispersion vs. longitudinal speed *p* = 0.05, speed dispersion vs. lateral speed *p* = 0.272 and longitudinal vs. lateral speed *p* = 1.00; and for the second half: speed dispersion vs. longitudinal speed *p* = 0.11; speed dispersion vs. lateral speed *p* = 1.00 and longitudinal vs. lateral speed 8 *p* = 1.00. The significant differences of length and width variables with the rest but not between them (for the first half *p* = 0.272 and for the second half *p* = 0.375), lead us to think that another intermediate timescale, which evolved over several seconds, could exist.

**Table 3:** **Multiple Comparisons ***p***-values (2-tailed) and Cohen's ***d*** results for (a) the first half and (b) the second half**.

**Positioning-derived variables**		**(a) First half**									**(b) Second half**							
		**1**	**2**	**3**	**4**	**5**	**6**	**7**	**8**		**1**	**2**	**3**	**4**	**5**	**6**	**7**	**8**
Stretch index	1	—								1	—							
Speed of dispersion	2	^*^vl	—							2	^*^vl	—						
Length	3	^*^l	^*^m	—						3	^*^l	^*^m	—					
Width	4	^*^l	^*^m		—					4	^*^l	^*^m		—				
Sectors	5		^*^vl	^*^l	^*^m	—				5		^*^vl	^*^l	^*^m	—			
Corridors	6		^*^vl	^*^l	^*^l		—			6		^*^vl	^*^l	^*^l		—		
Longitudinal speed of team center	7	^*^l	^*^s	^*^s	^*^m	^*^vl	^*^vl	—		7	^*^vl		^*^s	^*^m	^*^vl	^*^vl	—	
Lateral speed of team center	8	^*^vl		^*^m	^*^m	^*^vl	^*^vl		—	8	^*^vl		^*^m	^*^m	^*^vl	^*^vl		—

–*, Diagonal cell*.

* *p < 0.05*.

*Letters denote the magnitude: s, small; m, moderate; l, large; vl, very large*.

## Discussion

The current study explores the soft-assembly of tactical patterns and the timescales of positioning-derived variables that define them during a soccer match, allowing understanding the multilevel organization of tactical behaviors as defined by the timescales of evolution of collective patterns. For this goal, a hPCA and the dwell time measure were used to identify, respectively, nested correlated movement configurations as well as the characteristic timescales of their change (Hristovski et al., 2013). The main results afford that most stable/persistent movement patterns were well-related to defensive behaviors. In fact, team contraction, expressed through lower stretch index values and the drop-back of the team, are suggested as being key indicators of team behavior in the defensive phase (Bourbousson et al., 2010; Frencken et al., 2011). These results do not indicate that the team performed more defensive patterns, rather the observed patterns are likely to be most stable (Travassos et al., 2011; Ric et al., 2016). The degree of similarity between the PC2 extracted for both halves elucidates that the offensive playing style of the team is clearly defined. In this sense, it can be said that independently of the players, the playing style proposed by the coach strongly constrained the emergence of concrete tactical behaviors. These patterns, which explain the larger percentage of variance, describe collective behavior when the team or the opponent perform long ball possessions, by stabilizing their behavior and during which small reconfigurations take place. Notwithstanding, some PCs also exhibit some patterns that corresponded to game transitions (losing or winning back the ball; Sarmento et al., 2013). For example, PC12 in the first half and PC5 in the second defined counterattacks by the team analyzed. The small stretch index was quickly expanded and the team was moving rapidly forward starting from the mid-defensive sector. However, it is possible to detect some tactical patterns defined by a pressing strategy to win back the ball by using player distances from their team geometrical center and its depth in respect of goal (Frias and Duarte, 2014). In this hypothetical case, the team geometrical center would move forward while team dispersion would reduce in size. This structure of PCs could be related with PC7 and PC8 in the first half and with PC6 and PC9 in the second.

Previous findings revealed that depending on the situational context of the match the teams switch between different functional states (Frencken et al., 2012). The results have shown those of specific tactical patterns brought about by the influence of key events, like goal-scoring (Lago-Peñas and Dellal, 2010). Figure 2 allows identifying the types of patterns that appeared at different game moments. In this sense, the goal was preceded by a counterattack by the opposing team. Consequently, coaches should consider training specifically those patterns which might lead to conceding/scoring a goal. Even so, such defensive patterns are obviously preceded by an attacking phase, then ball recovery strategies and the context in which this would happen must be considered in the training task designed (Barreira et al., 2014).

In this study the hypothesized soft-assembled multilevel dynamics (Hristovski, 2012; Hristovski et al., 2013) in a soccer match have been corroborated. The salient correlated patterns (PCs) in the first level of analysis revealed tactical behaviors on higher levels. This finding helps to understand the nested organization of tactical behavior. At the highest level, a sole collective variable (PC) for each half exhibits the essence of tactics. The levels evolve on different timescales and only the tactical variables that evolved over longer timescales significantly contributed to the structure of highest-order PCs. That is, the speed of team geometrical center evolves on a scale of seconds, the stretch index (player dispersion) remains below 16.75 m during more than 20 s on average (timescales of tens of seconds), before switching expansion, and vice versa. Therefore, the hierarchy is a consequence of the correlated lower-order PCs. The most time-persistent categories (those with long dwell times) create correlations between the lower-order PCs. This was revealed in the highest order PCs where two out of three most time-persistent categories were those that had by far the highest scores. In summary, whereas the highest level that captures the essentials of team tactical behavior corresponds to slower changes (a few tens of seconds), the lowest order of PCs quickly evolve, being more susceptible to the sensitive changes of constraint-induced actions. It is important to note that this emergence of tactical behaviors in soccer as in other team sports results from reciprocal influences (bottom-up, top-down) of performer-environment interactions on different timescales (Hristovski et al., 2011).

Although previous studies have demonstrated that the average values of these positioning-derived variables were significantly different between both halves and also dependent on ball possession (Clemente et al., 2013), the dwell times of most of these variables were not significantly different comparing the first and second halves. This invariance in the timescales has given rise to the hierarchical structure of tactical patterns in both halves being mostly the same. However, the additional level identified in the second half suggests that tactical patterns carried out by the second group of teammates performed more varied behaviors because of the less correlated behaviors in the second-level of principal components. In spite of this, the principal component extracted in the highest-level of both halves reached a high value of similarity. This congruence in score distribution suggests that independently of the group of teammates, the essence of team tactics was similar during the match.

These findings open the way to analyzing how teams behave during a competition and studying the influence of different constraints (e.g., score, substitutions, instructional constraints, opponent level, etc.) over the course of the match. Furthermore, due to the limitation of the unknown opponent and ball position, more research is needed to ascertain the soft-assemble action landscape of different teams and to compare the collective tactical patterns of two confronted teams. The positioning of the opponent would allow analyzing the coupling between the teams' behavior, and to detect if some of them lead pattern-forming. The results should be balanced considering that only one match was used to capture the tactical behaviors. Despite this limitation, this study leaves an open path to exploring match-to-match invariant behaviors. In addition, this approach warrants further investigations on using ecological task constraints during training to develop the potential landscape of tactical patterns allowing the spontaneous emergence of novel modes of coordination and/or specific tactical performance solutions. This study also provides the possibility to determine relevant timescales for scoring goals and shooting at goal using a large goal-scoring sample.

## Conclusion

This approach can help to identify tactical patterns during different matches, comparing the degree of similarity between them. Further analysis would allow determining the influence on team tactical behaviors of different situational variables, e.g., rank of opponent, score of the previous match, playing home or away, which remain invariant during a longer timescale, i.e., days. Moreover, this analysis can help coaches to verify if the essence of tactics or playing style performed during training sessions is definitively developed during competition and if it remains invariant from match to match. Moreover, it would allow coaches to optimize training drills developing their style of play and/or identify the tactical behaviors performed during the match to design a representative training task. Finally, the characteristic timescales of collective behaviors allow understanding the formation of the hierarchically nested structure of tactical patterns in an ecological context and presents a rationale to define soft-assemble multilevel dynamics in soccer matches.

## Author contributions

AR worked on the design of the study, collection, analysis and interpretation of data, and drafting the manuscript. CT participated in the conceptualization and design of the study, data collection and reviewed the content of the manuscript. BG worked on the design of the study, data collection, statistical analysis, and reviewed the content of the manuscript. JS participated in the conceptualization and design of the study and reviewed the content of the manuscript. RH conceived the approach to data analysis, data interpretation, and drafting of the manuscript. The authors approved the final version and agree to be accountable for all aspects of the work.

## Funding

This study is supported by the National Institute of Physical Education of Catalonia, Generalitat de Catalunya.

### Conflict of interest statement

The authors declare that the research was conducted in the absence of any commercial or financial relationships that could be construed as a potential conflict of interest.

## Footnotes

^1^Over time several models were proposed that can account for metastability: Stochastic hopping within the enduring potential landscape (a simple bistable potential scenario Eyring, 1935; Freidlin and Wentzell, 2012) or a much more involved quenched disorder scenario (Cugliandolo and Kurchan, 1993), hopping due to the changing stability of the fixed point attractors (minima become maxima and vice versa, i.e., an annealed disorder scenario Badii and Politi, 1999), transient dynamics in a homoclinic channel (Kelso, 1995), and transient dynamics in a stable heteroclinic channel (Rabinovich et al., 2008) as well as the self-organized criticality scenario (Bak et al., 1987; Kello et al., 2008). All these models claim descriptions of metastability. However, they differ. Quenched and annealed disorder models differ in the timescales ascribed to the interactions (constraints) among the dynamic degrees of freedom of the system. Both systems are multistable in the sense of having a vast number of well-defined fixed point attractors i.e., minima. While in the quenched systems interactions are assumed to be to a good approximation constant with respect to the dynamical degrees of freedom, in annealed disorder systems they change on the same timescale. Hence the difference in the behavior of these systems: enduring vs. changing potential landscapes. These two models are stochastic in a sense that the interactions between the dynamic degrees of freedom are random. Real complex systems, such as teams of players, may even be a combination of both these polar types, showing both types: constant and quickly changing interactions. This would produce landscapes with some enduring and some changing basins of attraction. Contrary to these models, homoclinic and heteroclinic channel metastability are purely dynamic and no stochastic influence is needed although, as was the case in the heteroclinic channel scenario, noise, i.e., stochasticity, can be present. The self-organized criticality type of metastability arises as a domino effect, upsetting minimally stable states. On the other hand the data collected on the observational timescale enables the calculation of the probabilities of configurations. This further enables the presentation of the configurational probabilities as coming from the well-known statistical mechanics relation (via the maximum entropy method) between configuration probabilities and minima depths. Of course, such a depiction of probabilities, taken on the observational timescale, makes whether this quicker dynamics comes from the enduring or changing landscape or any other form of quick dynamics irrelevant. The enduring landscape scenario, in this case, becomes a useful tool to represent the dynamics of the configurations (see Hristovski et al., 2011; Ric et al., 2016). Of course, alternative models may exist as is the case in many areas of science. Here we find the scenario of hopping dynamics on an enduring landscape as being the most parsimonious at present, although a mixed quenched-annealed disordered scenario would be rather more realistic. Bovier and Den Hollander (2016) define metastability in a model-independent way as a property related to the existence of multiple separated timescales. At short time-scales, the system appears to be in equilibrium, but in fact, explores only a limited part of its available state space. At longer timescales, it undergoes transitions between such metastable states. This definition satisfies the models with enduring and changing potential landscapes as well as the homoclinic and heteroclinic channel models of metastability. However, it does not satisfy the metastability of the self-organized criticality type in which timescales are not separated and independent. This example shows that the definition of metastability is somewhat flexible. Nevertheless, in all of these models there exist at least two timescales (for convenience called quick and long-term).
